# NEURONAL-SPECIFIC PROTEASOME AUGMENTATION VIA EXTENDS LIFESPAN AND REDUCES AGE-RELATED NEURODEGENERATION

**DOI:** 10.1093/geroni/igz038.364

**Published:** 2019-11-08

**Authors:** Andrew M Pickering

**Affiliations:** UT Health San Antonio, San Antonio, Texas, United States

## Abstract

Cognitive function declines with age throughout the animal kingdom and increasing evidence shows that disruption of the proteasome system contributes to this decline. The proteasome has important roles in multiple aspects of the nervous system, including synapse function and plasticity, as well as preventing cell death and senescence. We report that augmentation of proteasome function, using overexpression of the proteasome β5 subunit, enhances proteasome assembly and function. Significantly, we go on to show neuronal-specific proteasome augmentation slows age-related declines in measures of learning, memory, and circadian rhythmicity. Surprisingly neuronal specific proteasome augmentation of proteasome function also produces a robust increase of lifespan in Drosophila melanogaster. Our findings appear specific to the nervous system; ubiquitous proteasome overexpression increases oxidative stress resistance but does not impact lifespan and is detrimental to some healthspan measures. These findings demonstrate a key role of the proteasome system in brain aging.

